# Supplementary figures and images for: Identification of host lncRNAs that impact Venezuelan equine encephalitis virus replication
Source: bioRxiv. 2025 May 12:2025.05.12.653438. Preprint. [Version 1] doi: 10.1101/2025.05.12.653438 (PMC12132526; doi:10.1101/2025.05.12.653438)

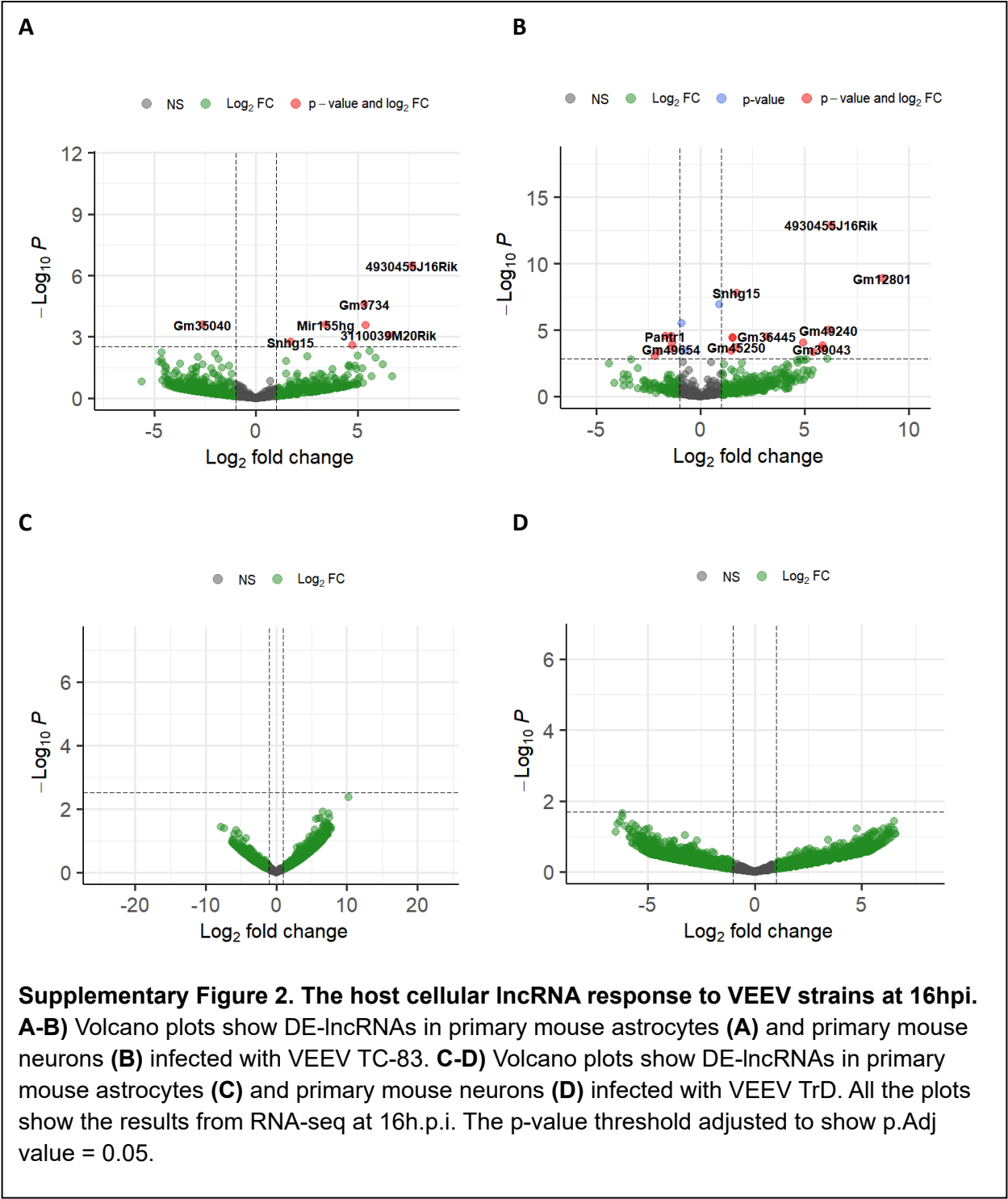

587

588

589

Supplement: Supplement 1 [file NIHPP2025.05.12.653438v1-supplement-1.pdf]
